# Trial management– building the evidence base for decision-making

**DOI:** 10.1186/s13063-017-2322-8

**Published:** 2018-01-08

**Authors:** Shaun Treweek, Roberta Littleford

**Affiliations:** 1 Health Services Research Unit, University of Aberdeen, Aberdeen, UK; 2 Tayside Clinical Trials Unit, Tayside Medical Science Centre, University of Dundee, Dundee, UK

## Abstract

Once the excitement of trial design, grant-writing and award are behind us, the great open expanse of the next few years is filled almost exclusively by trial management, the nitty-gritty of getting stuff done – delivering the goal. The most important members of the trial team now are not the professors and investigators but the trial managers. These trial managers have limited published information to help them make informed decisions about how to handle the day-to-day challenges that trials present. This special series aims to highlight the fact that writing on trial management is important, publishable and that *Trials* would welcome more of it.

## Editorial

In many ways a trial is like a small business. There are clearly products – the new knowledge about a treatment that the trial will generate, evidence to support practice and potentially a new or improved approach to treatment, and better health outcomes for patients. But there are also staff to hire, train and manage, regulations and laws to adhere to, finances to manage, contracts, procurement, paperwork and reporting and, of course the customers – the trial participants – who you need to buy-in to the study and who you want to stay loyal to your trial brand. All of this may have to be sustained for years.

Unlike the heaving shelves of bookshop business and management sections, the trial management bookshelf is remarkably untroubled by published material. There is a skewed supply and demand dynamic – high demand but minimal supply: “Managing clinical trials” by Barbara Farrell, Sara Kenyon and Haleema Shakur and published in *Trials* in 2010 [1] has had almost 100,000 accesses to date and is the second most accessed article of all time in *Trials*.

Once the excitement of trial design, grant-writing and award are behind us, the great open expanse of the next few years is filled almost exclusively by trial management, the nitty-gritty of getting stuff done – delivering the goal. The most important members of the trial team now are not the professors and investigators but the trial managers. These trial managers have limited published information to help them make informed decisions about how to handle the day-to-day challenges that trials present. There are networks that provide specific training and support, for example the Trial Managers’ Network (http://www.tmn.ac.uk) in the UK and the Association of Clinical Research Professionals (https://www.acrpnet.org) and Society of Clinical Research Associates (http://www.socra.org) in North America. There are also some tools to help (see for example http://www.ct-toolkit.ac.uk/routemap/trial-management-and-monitoring/) and the European Clinical Research Infrastructure Network, ECRIN (http://www.ecrin.org) is mapping international trial infrastructure support, including trial management. There are professional management qualifications that trial managers could seek. But examples of writing about effective trial management approaches, empirical evaluations of different approaches to management and decision-making, how and if trial management should affect trial design, and conduct and what we can learn from other management disciplines and from other studies are sadly lacking. There is limited sharing of lessons learned, of how problems were solved. This hinders trial managers’ ability to move their discipline forward and may be reducing trial efficiency.

This special series aims to highlight the fact that writing on trial management is important, publishable and that *Trials* would welcome more of it because one product that isn’t being delivered from the multitude of small businesses that trials represent, is an increase in shared knowledge about effective trial management. This must change. All of us in trials need to think about ways in which we can make this knowledge available to others, including perhaps new types of publication. This series will, we hope, start to provide some supply for all that demand.
